# Supplementary material for: Seroprevalence of SARS-CoV-2 and risk factors for infection among children in Uganda: A serial cross-sectional study
Source: PLoS One. 2024 Dec 23;19(12):e0312554. doi: 10.1371/journal.pone.0312554 (PMC11665985; doi:10.1371/journal.pone.0312554)
Supplement: Attachment — Submitted filename: Response_to_Reviewers.docx [file pone.0312554.s002.docx]

Reviewers' comments:

5. Review Comments to the Author

Please use the space provided to explain your answers to the questions above. You may also include additional comments for the author, including concerns about dual publication, research ethics, or publication ethics. (Please upload your review as an attachment if it exceeds 20,000 characters)

Reviewer #1: This is a manuscript on an important subject of COVID 19 and prevalence risk factors and effect on vulnerable population. It is well written but there are several clarifications that needs to be done to make it even better.

-Please clarify who is the Corresponding author is. Is it Jessica Briggs or Irene Bagala?

**I have updated this to reflect that it is Jessica Briggs after discussing with Irene.**

-Since you have not reported testing The SARS COV2 strains, you can only conclude that the seroprevalence --was 'during' the said strain was circulating and not caused 'by' the said strains

**-Changed the word "by" to "during" in the abstract (line 47) and in the Discussion, (line 280).**

-Not clear how the 12 and the 32 communities were picked out of 64 MRC

**Funding was only available to test samples from 12 of the communities at baseline. Before the follow-up survey, additional funding was obtained and the protocol was amended to allow us to survey all ages in 32 of the 64 communities instead of children 2-10 only. There were 2 MRCs per district, so 1 MRC from each district was chosen for the all-ages survey. Only samples from children aged 0-17 were tested in the follow up survey as detailed in the methods. Edited line 120 in the methods to reflect the protocol modification and line 122 to explain that one cluster was chosen per district.**

-Not clear why the consent to use stored samples was waived yet you had access to the population ( what reasons did you give the ETHICS review board?)

**During the initial collection of samples for the LLINEUP2 cross-sectional surveys, participants could consent to "future use of biological samples." Therefore, we were only allowed to test samples from those patients who had consented to future use of the dried blood spots collected. Even so, we had to obtain an additional waiver from SOMREC to perform this COVID-19 sub-study using these de-identified samples, as detailed in lines 180-181.**

-I feel the word “Impact” is too strong, could you replace with another word such as “effect”

**I am unsure which use of the word "impact" the reviewer is referring to. If it is the use of it in the abstract, I would argue that "effect" is a synonym for "impact" and many papers that we cited (see references) even use the term impact in their titles. (eg References 2, 32, and 34).**

-You introduce the use of samples from other studies without giving a short brief on the studies.

**Lines 142-143 have been edited. The negative controls were obtained from a cohort study conducted before the COVID-19 pandemic, and the positive controls were obtained from participants with PCR-confirmed SARS-CoV-2 infection in a study at UCSF. Also, these studies are referenced, so that the reader can find the original studies if interested.**

-At baseline you do not have results for under 2 years- any reason?

**This was a sub-study conceived after the LLINEUP2 randomized controlled trial had started; the original version of the trial protocol did not allow for collection of samples in children <2 years. We were able to modify the protocol to collect samples from children <18 years in time for the follow-up survey.**

-Please state if an In-house determination of cut of using R package ROCR would affect comparability with other studies?

**Comparability to other studies would be affected not by the use of ROCR which uses standard formulas to calculate sensitivity and specificity, but by the characteristics of the Luminex assay and the positive and negative controls used. Therefore, we compared our results to other published SARS-CoV-2 serologic data in Uganda (lines 296-301) and found that our results were consistent with seroprevalence studies conducted in Uganda in other age groups.**

Reviewer #2: Bagala et al. describe the results of a cross-sectional survey nested in a cluster-randomized trial conducted in Uganda. This report informs children’s exposure to SARS-CoV-2 across epidemic waves while analyzing knowledge, attitudes, and behavior toward COVID-19 at the household level. Overall, the manuscript is well-written and presents relevant data. However, the following points need to be clarified/improved:

Background
L56. “SARS-CoV-2 acronym already includes virus in it. So, there might be no need to add “virus” after SARS-CoV-2.

**Fixed on line 58.**

Methods
L99-100. “For each cluster for each survey…” Could the authors rephrase to avoid repeating “each”?

**Fixed, line 104-105.**


L111. Authors might need to rephrase as follows for a better understanding of the sentence: “… prick blood sample was collected from all children aged 2-10 present in the household.”

**Fixed lines 115-116**

L116-117. “…along with assent if the child was 8 years old or older…” could be replaced with “…along with assent if the child was at least eight years of age or at least eight years old”.

**Done, lines 124-125.**

L160-164. The authors state that four institutions approved the study. Yet, they didn’t provide approval numbers. “LLINEUP2” seems to be the project name, and other details provided in L160 refer to the clinical trial registration of the main study.

L190. Please add the content in parenthesis rather after “study” than after “The parent.”

**Fixed; lines 171-176, and also in the ethics statement in the declarations.**

Results
A general concern is that the authors mention in the abstract and methods that they adjusted the seroprevalence to account for clustering within households and for the test kit performance. Could they provide crude seroprevalence estimates to see whether adjustments significantly changed the figures?
**Raw seroprevalence at each survey timepoint can be calculated from Table 2, but I have also added these figures into the text at lines 249-251. As you can see, the adjustment for characteristics of the assay results in minor changes to the raw seroprevalence figures.** **The effect of household clustering was adjusted for only in the logistic regression models used to generate the results in Table 2.**

L175, 179, 180. I wonder why Figure 2 appears before Figure 1 while Figure 1 appears for the first time in the Results section.

**Fixed, and figures re-labeled correctly.**

L188. “…the children were ages…” This is likely a typo, please correct.
**Fixed, line 205**

L192 vs L193. Are the authors presenting data on attitudes or perceptions? Both seem to be used interchangeably in the next paragraphs.

**The word "perception" was only used once; it has been changed to "attitude" on line 210.**

L175-226. Although data on knowledge, attitudes, behaviors, and vaccination are important to understanding COVID-19, they seem to draw the reader’s attention more than the actual seroprevalence data, which is supposed to be the focus of this study. Presenting seroprevalence data first to address the main research question and then mentioning additional data could be one way forward.

**Changing the order of the results per this reviewer's opinion would require restructuring of the paper. Because this was a concern brought up by only one of three reviewers, and not by any of the co-authors upon review of the paper, we would prefer to keep the results as they are presented currently.**

L295-297. Please rephrase the sentence for a better understanding.

**Fixed, lines 315-319.**

Figure 3. Please indicate the north and the scales on the provided maps of RBD seroprevalence at baseline and follow-up

**Added north and scales on new Figure 3.**

L373-377. Please provide the approval number for the listed ethical approvals

**Done as stated above.**

Reviewer #3: This is a straightforward analysis of a sub-study conducted within the context of a large, cluster-randomized trial. The statistical analysis is appropriate and well-conducted, although I would like to see an enumeration of the variables included in the multivariate models. The manuscript is clearly written, with cogent discussions of study limitations. I have little to recommend in the way of changes. I might quibble over whether households' omitting fomites as a SARS-CoV-2 transmission truly represents a lack of knowledge (I'm not sure fomites have ever been shown to be even a moderate exposure risk), but this is minor and debatable. The results have modest immediate utility, but assessing "importance" is beyond the scope of review. In any case, as the largest seroprevalence survey of children in Uganda to date, the study is valuable.

**Variables included in the multivariate models can be found in Table 2.**
